# Modeling and Analysis of Micro Surface Topography from Ball-End Milling in a Trochoidal Milling Mode

**DOI:** 10.3390/mi12101203

**Published:** 2021-09-30

**Authors:** Yongheng Dong, Shujuan Li, Qian Zhang, Pengyang Li, Zhen Jia, Yan Li

**Affiliations:** 1 School of Mechanical and Instrument Engineering, Xi’an University of Technology, Xi’an 710048, China; DongYongheng@xaut.edu.cn (Y.D.); lipengyang@xaut.edu.cn (P.L.); 1190210001@stu.xaut.edu.cn (Z.J.); jyxy-ly@xaut.edu.cn (Y.L.); 2 School of Mechanical and Electrical Engineering, Guilin University of Electronic Science and Technology, Guilin 541004, China; txpg8405@163.com

**Keywords:** ball-end milling cutter, trochoidal milling, micro surface topography, modeling, Z-MAP method, surface characterization

## Abstract

The trochoidal milling mode is widely used in high-speed machining, and due to good adaptability and flexible posture adjustment, ball-end milling cutters are conducive to complex surface machining with this mode. However, the processes of material removal and formation of machined micro surfaces are very difficult to describe as the profile of cutter teeth is complex and the trajectory direction changes continuously during the trochoidal milling process. A modeling method for the generation of micro surface topography of ball-end milling in the trochoidal milling mode is put forward. In this method, the locus equation of each cutter tooth is established based on the principle of homogeneous coordinate transformation, after which a Z-MAP algorithm is designed to simulate the micro surface topography. The Z-MAP algorithm can quickly obtain the part grid nodes potentially swept by the cutter tooth within a unit time step through the establishment of servo rectangular encirclement and instantaneous sweeping quadrilateral of the element of cutter teeth; the part grid nodes actually swept are further determined through an angle summation method, and the height coordinate is calculated with the method of linear interpolation according to Taylor’s formula of multivariate functions. Experiments showed that the micro surface topography resulting from ball-end milling in the trochoidal milling mode had high consistency with the simulation, which indicates that the proposed method can predict micro surface topography in practical manufacturing. In addition, a comparison of micro surface topography between trochoidal milling and ordinary straight-linear milling was conducted, and the results showed that the former was overall superior to the latter in resulting characteristics. Based on this conclusion, the influences of cutting parameters of ball-end trochoidal milling on surface characteristics, particularly amplitude and function, were analyzed according to the simulated micro surface topography data.

## 1. Introduction

Ball-end milling is a very adaptable process which is widely used in the machining of complex parts for the aerospace, automotive, die and mold industries. The micro surface topography of machined parts after ball-end milling has a significant impact on the product performance. Ball-end milling using the trochoidal milling mode (i.e., Ball-end trochoidal milling) can improve surface performance. Modeling and simulating the micro surface topography is beneficial to analyze the means by which cutting parameters influence it, and to provide a basis for optimizing cutting parameters.

Traditionally, the vibration assistant method is widely used in the milling processing, and its trochoidal trajectory is beneficial to improve milling performance and surface quality [1,2,3]. Specialized add-on devices, however, are required in this method, resulting in high cost and difficulty in control. With the development of precision controlled technology, a trochoidal milling trajectory can be directly determined by the numerical controlled system of a milling machine, and this trochoidal milling mode can be used to machine difficult-to-machine materials and cavity parts due to its low cutting force, high surface quality and high productivity [4]. Trochoidal milling mode refers to milling parts using a trochoidal trajectory [5]. Uhlmann et al. [6,7,8] applied this mode to machine TiAl6V4 and nickel-based super alloy Inconel Alloy 738, and found that it could improve the machinability of difficult-to-cut materials, reduce machining energy consumption and improve machining efficiency. Ferreira et al. [9,10] investigated the validity of trochoidal milling on machining islanding and cornered cavities, finding that trochoidal milling mode could improve the machining conditions of cavity parts and significantly increase efficiency. Zhang et al. [4,11] established a model for trochoidal milling force involving the radial depth, entry and exit angle of cutter teeth, and found that the proposed model, compared with the traditional milling method, reduced the instantaneous acting area between the cutter and the part, and thus reduced the cutting force. However, all of the above research mainly focused on the feasibility of trochoidal milling for difficult-to-cut materials, and on the reduction in cutting force it can provide for complex cavities, with few reports examining the resulting micro surface topography.

An important aspect of surface quality, micro surface topography refers to the irregular micro geometry such as surface roughness, waviness, shape error, and texture [12], which not only affects surfaces’ wear, lubrication state, friction coefficient, etc., but also other properties of the parts’ contact state [13,14,15]. Trochoidal milling has complex trajectories and consequently forms micro surfaces via complicated mechanisms. A suitable micro surface topography modeling method is required to reveal the forming mechanisms and rules associated with micro surface topography. The machined micro surface topography of parts mainly depends on the final process. When precision milling is used as final machining, a high spindle speed, small feed per tooth, and small cut depth far from the self-induced vibration (chatter) threshold is commonly adopted. This strategy generates little milling force, especially when milling easy-to-machine materials (such as aviation aluminum alloy, etc.). Chatter could be effectively decreased if the process is sufficiently rigid, and the commonly adopted strategy of high-speed milling could prevent the generation of built-up edge and scale thorn [16], with its strong adiabatic shear [17] reducing the plastic lateral flow of the instantaneous machined material layer. Therefore, the geometric characteristics of the cutter, trajectory design and cutting parameters are the main factors which affect the final micro surface topography. Precision milling surface quality can be controlled by establishing a geometric simulation model in order to predict the micro surface topography.

A common method for modeling micro surface topography, the Z-MAP algorithm isolates the bottom plane of the part and converts it into x-y grid nodes, then calculates the corresponding z-coordinate of each grid node according to the position of the cutter teeth in the cutting process, and finally describes the machined surface with an x-y-z vector [18]. Compared with other main simulation methods, such as solid modeling based on Boolean algorithms [19,20,21] and the ’bridge’ method [22,23], the Z-MAP algorithm overcomes the disadvantages of both: the former only considers the residual height of the machined surface in the interval milling feed direction and ignores that in the feed direction, and the latter reduces computational difficulty by analytical methods, such as numerical calculation or approximation, to solve the residual height. In addition, the Z-MAP algorithm can consider various factors influencing the micro surface topography, and thus produces simulation results closer to the actual results compared with the other methods. Therefore, researchers have applied Z-MAP to simulate micro surface topography obtained by various processes such as turning [24], grinding [25] and milling. In milling simulation, Hu et al. [26] studied the influence of cutter assembly error on machined surface topography using the peripheral milling process, Li et al. [27] researched surface machining accuracy in high speed peripheral milling of a flexible system, Dang et al. [28] put forward a method for prediction of surfaces resulting from peripheral milling based on an arc approximately sweeping the trajectory, Arizmendi et al. [29] modeled surface topography resulting from peripheral milling taking tool vibration into consideration, Schmitza et al. [30] investigated the effect of milling cutter teeth runout on surface topography in peripheral milling, Omar et al. [31] predicted end milling surface topography, Gao et al. [32,33] proposed a method for the prediction of peripheral milling surface topography combined with a numerical simulation method, and Franco et al. [34,35] built a simulation model for surface topography resulting from round corner milling cutters and face milling cutters. However, less attention has been paid to simulation models based on ball-end milling cutters, which have a more complex profile of the cutter teeth. With the maturing of multi-axis control technology, precision milling with a ball-end milling cutter can replace grinding (or polishing) and directly reach the required quality of complex surfaces and profiles. Predicting micro surface topography through modeling and simulation can provide a scientific basis for the selection of processing parameters. Liu et al. [36,37,38,39] established an extendable simulation model for ball-end milling micro surface topography, in which factors such as wear and orientation adjustment of the cutter were considered. These studies, however, focused on the acquisition of micro surface topography under linear trajectory milling without considering complex trajectories such as trochoidal tracks. The key utility of the Z-MAP algorithm is its ability to calculate the height coordinates of the discrete part grid nodes. Bouzakis et al. [40,41] employed the weighted mean interpolation method along with the plane approximation method to calculate the height of the micro surface topography at non-sampling time, which had fast computation time but easily caused approximation errors. Liang et al. [18,42,43] used a numerical iterative method for solving nonlinear equations to acquire micro surface topography using a high precision but complicated calculation process.

In order to model and analyze the micro surface topography of ball-end milling using the trochoidal milling mode, a Z-MAP modeling method is proposed. Based on the principle of homogeneous coordinate matrix transformation, a cutter tooth trajectory equation for ball-end trochoidal milling is established, after which the Z-MAP method combined with the time step method is proposed to simulate the micro surface topography. In this method, once the Z-MAP model of the part is established, the micro elements of cutter tooth and time step are set scientifically to ensure that the cutter tooth element can sweep no more than one workpiece grid point per time unit; subsequently a rounding method is used to determine a suspected grid node of a part which is close to the discrete point of the cutter tooth, and a nonlinear equation using Taylor expansion is used to solve the height coordinates of the suspected grid node. If the current height coordinates are lower than the original ones, the material above the grid node is presumed to be cut by cutter tooth elements and the original height coordinates are replaced by the current ones, and the micro surface topography is obtained. On the basis of simulation results, not only could micro surface topography comparisons between trochoidal and linear milling be conducted, but also four amplitude parameters and four functional parameters were used to analyze the influence of the cutting parameters of ball-end trochoidal milling on surface characterization.

The outline of the paper is as follows: In Section 2, the motion trajectory equation of a ball-end milling cutter tooth is established separately from the trochoidal trajectory of the cutter, with the posture taken into account. In Section 3, a method for simulating ball-end trochoidal milling micro surface topography is proposed based on the Z-MAP algorithm. In Section 4, the simulation effects are verified by vertical and milling experiments. Finally, the influence of cutting parameters on the micro surface topography and its characteristics are investigated by simulation.

## 2. Motion Trajectory Equation of a Ball-End Milling Cutter Tooth

**Figure 1:**
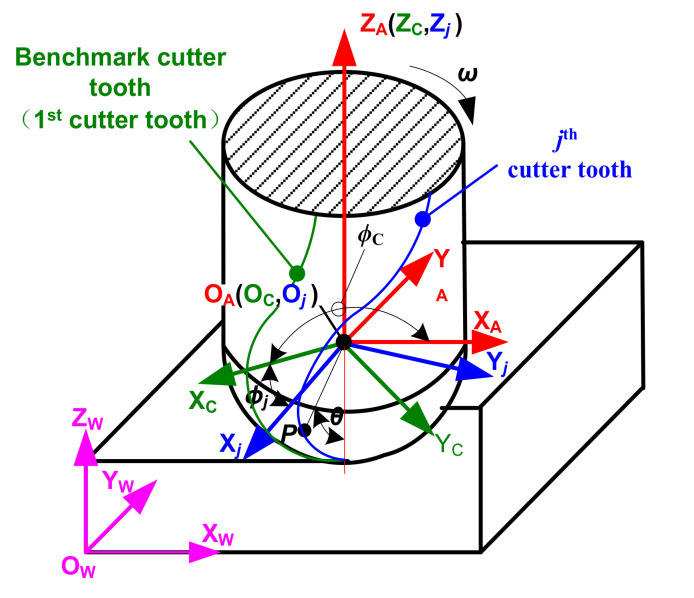
Cutter tooth trajectory reference coordinate system.

In order to establish the trajectory equation for an arbitrary cutting point on the cutter tooth during the trochoidal milling process of a ball-end milling cutter, rectangular coordinate systems are introduced as in Figure 1. The coordinate system O_W_-X_W_Y_W_Z_W_ is fixed on the part, and is therefore denoted the part coordinate system, {W} for short. The coordinate origin O_A_ of the coordinate system O_A_-X_A_Y_A_Z_A_ coincides with the center of the cutter’s ball head, and coordinate axes O_A_X_A_ and O_A_Y_A_ are parallel to coordinate axes O_W_X_W_ and O_W_Y_W_. Since the coordinate system moves (not rotates) with the spindle of the machine tool, it is denoted the spindle follow-up coordinate system, {A} for short. The coordinate system O_C_-X_C_Y_C_Z_C_ is fixed on the cutter; its coordinate origin O_C_ coincides with the center of the cutter’s ball head, coordinate axis O_C_Z_C_ coincides with the cutter’s center axis, and coordinate axis O_C_X_C_ passes the benchmark tool tooth (first cutter tooth). Since this coordinate system rotates around the spindle of the machine tool at the angular velocity of *ω* (rad/s), it is denoted the cutter coordinate system, {C} for short. The coordinate origin O*_j_* and coordinate axis O*_j_*Z*_j_* of the coordinate system O*_j_*-X*_j_*Y*_j_*Z*_j_* coincide with the coordinate origin O_C_ and coordinate axis O_C_Z_C_ of {C}, and coordinate axis O*_j_*X*_j_* passes the *j*th tooth; the system is therefore denoted the cutter tooth coordinate system, {*j*} for short, and the number of {*j*} is equal to the total number of cutter teeth.

For simplicity, this paper only considers a ball-end milling cutter with plane teeth, and the coordinates of an arbitrary point *p* on *j*th tooth in {*j*} are

(1) $$\left[x_{jp},y_{jp},z_{jp},1\right]=\left[R\sin\theta ,0,-R\cos\theta ,1\right]$$

where *θ* is the axial position angle (degree) of *p*, and *R* is the cutter’s radius (mm).

The transformation matrix of a ball-end milling cutter with multiple teeth from {*j*} to {C} is

(2) $$M_{Cj}=\left[\begin{array}{cccc} \cos\phi_{j} & -\sin\phi_{j} & 0 & 0\\ \sin\phi_{j} & \cos\phi_{j} & 0 & 0\\ 0 & 0 & 1 & 0\\ 0 & 0 & 0 & 1 \end{array}\right]$$

where *n_t_* is the total number of cutter teeth, and *ϕ_j_* is the radial position angle of *j*th cutter tooth (degree), equal to 360(*j* − 1) /*n_t_*.

**Figure 2:**
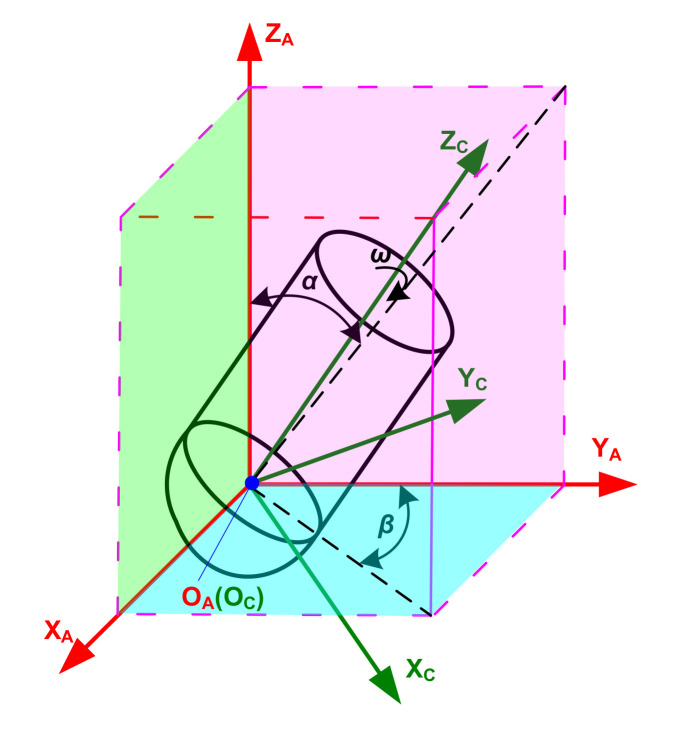
Adjustment of the cutter’s posture.

The adjustment of the cutter’s posture is mainly achieved through the lead and tilt inclination as shown in Figure 2, and the coordinate transformation matrices are

(3) $$R_{AX}=\left[\begin{array}{cccc} 1 & 0 & 0 & 0\\ 0 & \cos\alpha & -\sin\alpha & 0\\ 0 & \sin\alpha & \cos\alpha & 0\\ 0 & 0 & 0 & 1 \end{array}\right]$$

(4) $$R_{CY}=\left[\begin{array}{cccc} \cos\beta^{\prime } & 0 & \sin\beta^{\prime } & 0\\ 0 & 1 & 0 & 0\\ -\sin\beta^{\prime } & 0 & \cos\beta^{\prime } & 0\\ 0 & 0 & 0 & 1 \end{array}\right]$$

where *α* is the inclination angle of the cutter rotating around coordinate axis O_A_X_A_ in the feed direction (degree), which, called the tilt angle, is equal to the angle between the projection of coordinate axis O_C_Z_C_ and coordinate axis O_A_Z_A_ on the coordinate plane X_A_O_A_Z_A_; and *β* is the angle between the projection of coordinate axis O_C_Z_C_ and coordinate axis O_A_Y_A_ on the coordinate plane X_A_O_A_Y_A_ (degree), which is called the lead angle. In order to achieve the state shown in Figure 2, {C} is first rotated around coordinate axis O_A_Y_A_ to degree *β^’^*, which is equal to arctan(tan*β*cos*α*), and then {C} is rotated around coordinate axis O_A_X_A_ to *α*. Angles *α* and *β^’^* rotate counterclockwise in a positive direction, and clockwise in a negative direction.

Assuming that the angle between axis O_A_X_A_ and O_C_X_C_ is 0° at initial state, and spindle speed is *N* (rpm), {C} rotates around axis O_A_Z_A_ with angular velocity *ω =* π*N*/30 (rad/s), and the coordinate homogeneous transformation matrix is

(5) $$R_{CZ}=\left[\begin{array}{cccc} \cos(180\omega t/\pi ) & sin(180\omega t/\pi ) & 0 & 0\\ -\sin(180\omega t/\pi ) & \cos(180\omega t/\pi ) & 0 & 0\\ 0 & 0 & 1 & 0\\ 0 & 0 & 0 & 1 \end{array}\right]$$

Hence, the homogeneous transformation matrix from {C} to {A} is

(6) $$M_{AC}=R_{AX}R_{CY}R_{CZ}$$

**Figure 3:**
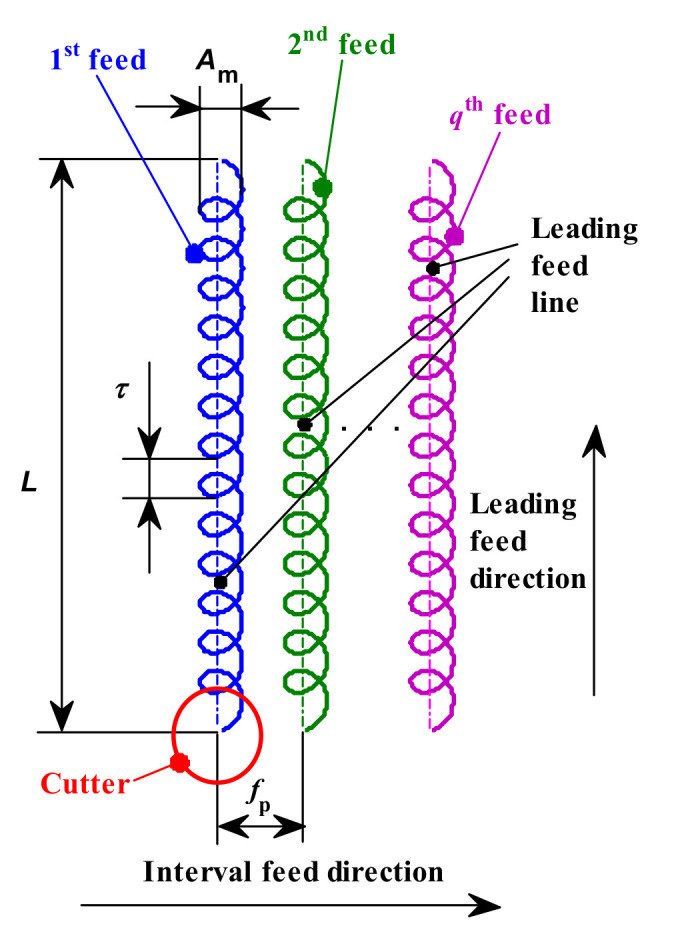
Feed trajectory of trochoidal milling.

Assuming that the leading feed is in Y direction, and the interval feed direction is in X direction, as shown in Figure 3. and assuming that the coordinate of cutter location O_C_ at start position o in {W} is $\left(x_{0},y_{0},z_{0}\right)$ in the first feed direction, the homogeneous transformation matrix from {W} to {A} is

(7) $$M_{\mathrm{WA}}=\left[\begin{array}{cccc} 1 & 0 & 0 & x_{0}+\frac{Nf_{f}n_{t}t[0.5A_{m}\sin\left(\eta \right)+\frac{\tau \eta }{360}]}{60\sqrt{0.25A_{m}{}^{2}+\frac{\tau \eta A_{m}\cos\left(\eta \right)}{360}+{\left(\frac{\tau \eta }{360}\right)}^{2}}}\\ 0 & 1 & 0 & y_{0}+(q-1)f_{p}+\frac{0.5Nf_{f}n_{t}tA_{m}\cos\left(\eta \right)}{60\sqrt{0.25A_{m}{}^{2}+\frac{\tau \eta A_{m}\cos\left(\eta \right)}{360}+{\left(\frac{\tau \eta }{360}\right)}^{2}}}\\ 0 & 0 & 1 & z_{0}\\ 0 & 0 & 0 & 1 \end{array}\right]$$

where *q* is the cutter feed number (*q* = 1, 2, 3...); *t* is the time from the start position of the *q*th feed direction to the current position (s); *f_f_* is the feed per tooth (mm/r); *f_p_* is the stepover between two adjacent leading feed lines (mm); *A_m_* and *τ* are the trochoidal amplitude (mm) and period (s), respectively; and *η* is the trochoidal polar angle (degree) and its range is [0, 360*L/τ*]. Trochoidal milling becomes linear milling when *A_m_* is equal to 0.

Based on the above transformation of the coordinate matrix, the trajectory equation for arbitrary point *p* on the cutter tooth in {W} is

(8) $${\left[x_{wp}\left(t,\theta \right),y_{wp}\left(t,\theta \right),z_{wp}\left(t,\theta \right),1\right]}^{T}=M_{\mathrm{WA}}M_{\mathrm{AC}}M_{Cj}{\left[x_{jp},y_{jp},z_{jp},1\right]}^{T}$$

## 3. Simulation Method for Ball-End Trochoidal Milling Micro Surface Topography

**Figure 4:**
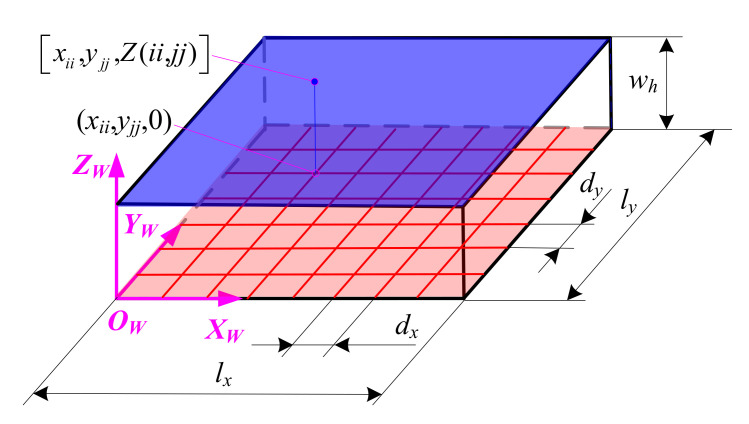
Z-MAP model of the part.

In order to simulate the micro surface topography produced by ball-end trochoidal milling, the part, cutter teeth and machining time need to be discretized. The part is represented by the Z-MAP model as shown in Figure 4, where a topological matrix *Z_iijj_* is introduced and the element *Z*(*ii*, *jj*) of *Z_iijj_* corresponds to Z coordinate value of a grid node (*ii*, *jj*) in the plane O_W_X_W_Y_W_, and where *ii* and *jj* are the numbers of the part grid node in the X and Y directions, respectively. The grid accuracy of the part is set as follows

(9) $$\max\left(dx,dy\right)\leq 1/5\min(f_{f},f_{p})$$

where, *d_x_* and *d_y_* are the discrete grid spacing of the part in the X and Y directions (mm), respectively.

**Figure 5:**
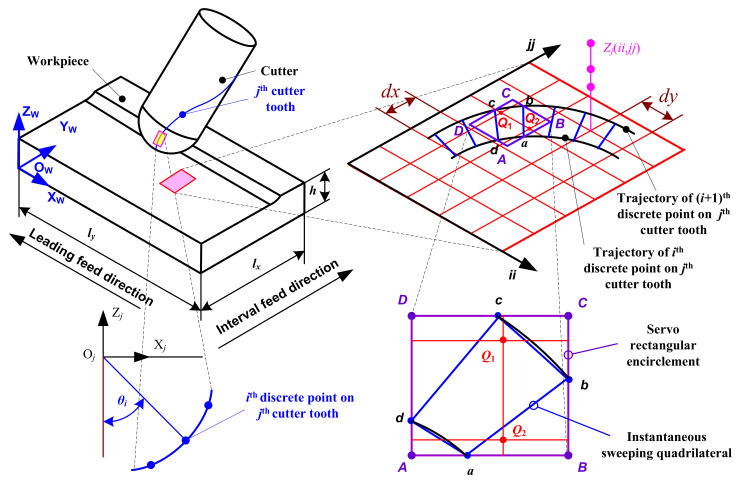
Ball-end milling micro surface topography generation principle.

**Figure 6:**
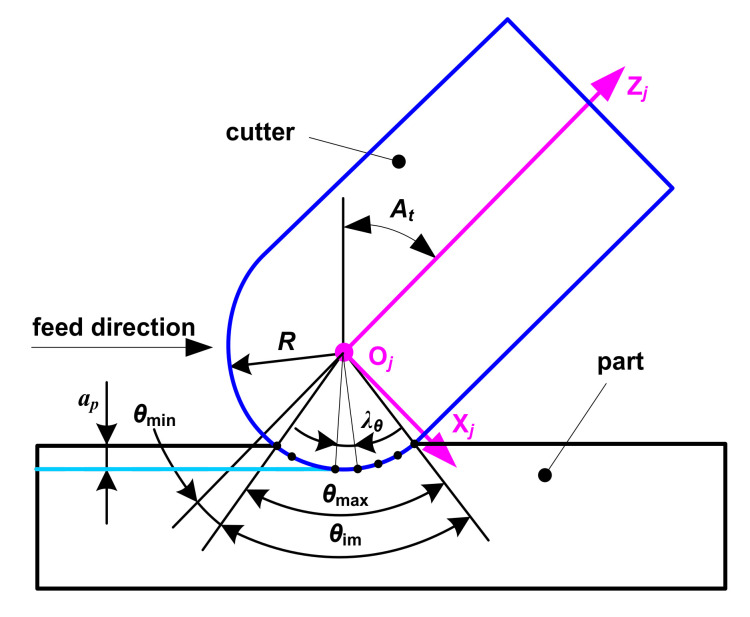
Cutter teeth microelement discretization.

The maximum projected length of discrete microelements of the cutter tooth in the plane O_W_X_W_Y_W_ cannot exceed the part grid spacing, to ensure calculating accuracy. In Figure 5, *θ_i_* represents the axial position angle of the *i*th discrete point on the cutter tooth, $\bar{dc}$ and $\bar{ab}\;$ are projection lines of the same discrete microelement at adjacent time intervals *t_ξ_* and *t_ξ +_*_1_, respectively, and the length of the projection lines are smaller than the minima of *dx* and *dy*. Considering the general working state of the cutter shown in Figure 6, cutter inclination *A_t_* (degree) and immersion axial position angle of the cutter tooth *θ*_im_ (degree) are calculated according to Equations (10) and (11), respectively. Minimum axial position angle *θ*_min_ (degree) and maximum axial position angle *θ*_max_ (degree) are equal to (*A*_t_ − 0.5*θ*_im_) and (*A*_t_ + 0.5*θ*_im_), respectively. If *θ*_min_ is lower than 0°, it is set to 0°; if *θ*_max_ is greater than 90°, it is set to 90°. The increment of axial position angle *λ_θ_* (degree) is given in Equation (12) so as to achieve the requirement mentioned above. *θ*_min_ and *θ*_max_ are assigned to the first and last values of *θ_i_*.

(10) $$A_{t}=\arccos\left(\cos\alpha \cos\beta \right)$$

(11) $$\theta_{\mathrm{im}}=2\arccos\left(\frac{R-a_{p}}{R}\right)$$

where *a_p_* is the cut depth (mm).

(12) $$\lambda_{\theta }=\frac{180\times \min\left(dx,dy\right)}{\pi R}$$

In terms of the discretization of machining time, the curves shown as $\overset{⏜}{da}$ and $\overset{⏜}{cb}$ in Figure 5 are the projected swept trajectory microelements of the cutter tooth’s discrete points during a unit time step *λ_t_* (s) on the plane O_W_X_W_Y_W_. The projected curves are assumed to be smaller than the minima of *dx* and *dy*. The discrete point closest to the outer edge of the ball-end milling cutter tooth, i.e., the point corresponding to the maximum axial position angle *θ*_max_ on the cutter tooth, sweeps the longest trajectory curve during *λ_t_*. Thus, based on the arc length of this curve, combined with the arc length formula of the curve calculated according to Equation (8), *λ_t_* is set as follows:

(13) $$\lambda_{t}=\frac{\min\left(dx,dy\right)}{\sqrt{x_{wp}{’}^{2}\left(t_{\xi },\theta_{\max}\right)+y_{wp}\prime^{2}\left(t_{\xi },\theta_{\max}\right)}}$$

In order to establish a servo rectangular encirclement and an instantaneous sweeping quadrilateral so as to quickly search the part grid node which is swept by the cutter tooth microelement in the current time interval, as shown in Figure 5, since *λ_t_* is small, $\overset{⏜}{da}$ and $\overset{⏜}{cb}$ are replaced by lines $\bar{da}$ and $\bar{cb}$, respectively. Points such as *a*, *b*, *c* and *d* are used as vertexes to establish an instantaneous sweeping quadrilateral of the cutter tooth microelement. The servo rectangular encirclement ABCD of the quadrilateral is established, in which sides CB (or DA) and AB (or DC) are parallel to the X and Y directions respectively. In a time interval, the part grid nodes falling into the servo rectangle encirclement can be judged from their X and Y coordinates in {W}, shown as *Q*_1_ and *Q*_2_ in Figure 5, which are suspected to be the swept points by the discrete cutter tooth microelement, and are therefore denoted the suspected fall-in points. The angle accumulating method is used to determine whether the suspected fall-in points are the real fall-in points or not.

Firstly, to connect one of suspected fall-in points with four vertices of an instantaneous sweeping quadrilateral respectively, and then calculate include angles between the two adjacent connecting lines according to Equation (14).

(14) $$A_{\kappa }=\arccos\left\{\frac{\left(x_{ii}-x_{u}\right)\left(x_{ii}-x_{v}\right)+\left(y_{jj}-y_{u}\right)\left(y_{jj}-y_{v}\right)}{\sqrt{\left[{\left(x_{ii}-x_{u}\right)}^{2}+{\left(y_{jj}-y_{u}\right)}^{2}\right]\left[{\left(x_{ii}-x_{v}\right)}^{2}+{\left(y_{jj}-y_{v}\right)}^{2}\right]}}\right\}$$

where $\left(u,v\right)\in \left\{\left(a,b),(b,c),(c,d),(d,a\right)\right\}$ correspond to *κ* = 1, 2, 3, 4, respectively, and (*x_ii_, y_jj_*) represent *x* and *y* coordinates of the suspected fall-in point (*ii, jj*) in {W}.

**Figure 7:**
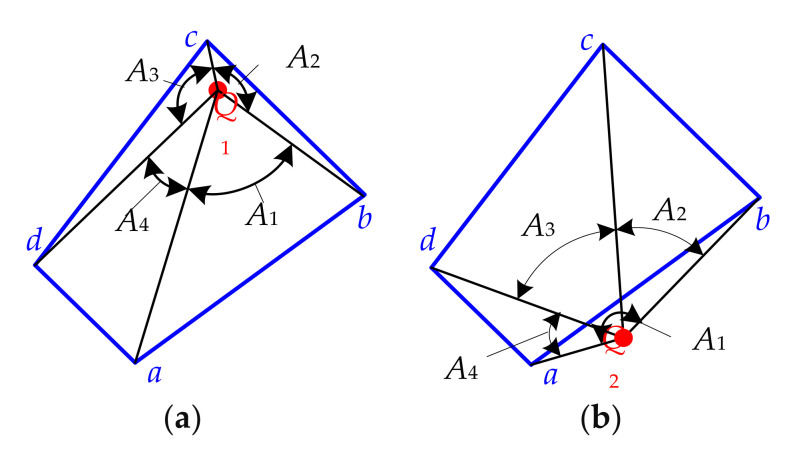
Schematic diagram of angle accumulation. (**a**) Fall-in point and (**b**) Not a fall-in point.

Secondly, to determine whether the suspected fall-in point actually falls into the instantaneous sweeping quadrilateral swept by the according discrete cutter tooth microelement through the angle accumulating method, it can be assumed to do so if *A*_1_ + *A*_2_ + *A*_3_ + *A*_4_ = 360°; otherwise, it does not. As shown in Figure 7, grid point *Q*_1_ is the fall-in point, and *Q*_2_ is not.

Thirdly, to judge which vertex of the instantaneous sweeping quadrilateral is the nearest one to the fall-in point, when the summation of two adjacent inclination angles is formed by three adjacent connecting lines between the vertexes, the fall-in point is closest to the largest, i.e., the vertex according to the middle connecting line is the closest to the fall-in point, and is called the calculating vertex p^*^. As shown in Figure 7a, (*A*_2_ + *A*_3_) is the largest, indicating that vertex *c* is closest to the fall-in point *Q*_1_, so *c* is the calculating vertex p*.

**Figure 8:**
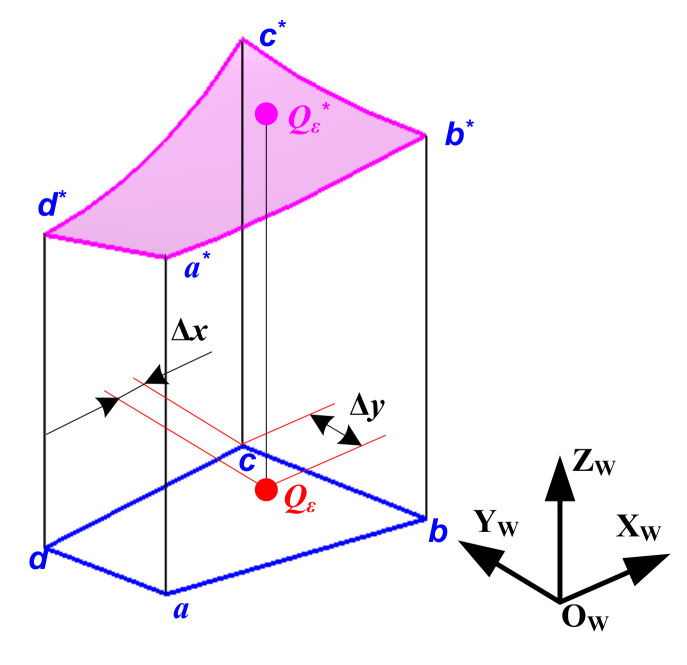
Diagram of height coordinates calculation in relation to the fall-in point.

To calculate height coordinates of the fall-in point, assuming that the X and Y coordinates of the calculating vertex p* in {W} are [*x_wp_*(t_ξ_*, θ_i_*), *y_wp_*(t_ξ_*, θ_i_*)], according to Equation (8), coordinates of the fall-in point *Q_ε_* are expanded out into Taylor’s formula

(15) $$\left\{\begin{array}{c} x_{ii_{Q_{\varepsilon }}}=x_{wp}(t_{\xi *},\theta_{i*})+\Delta x=x_{wp}(t_{\xi *},\theta_{i*})+\left(\Delta t\frac{\partial }{\partial t}+\Delta \theta \frac{\partial }{\partial \theta }\right)x_{wp}(t_{\xi *},\theta_{i*})\\ y_{jj_{Q_{\varepsilon }}}=y_{wp}(t_{\xi *},\theta_{i*})+\Delta y=y_{wp}(t_{\xi *},\theta_{i*})+\left(\Delta t\frac{\partial }{\partial t}+\Delta \theta \frac{\partial }{\partial \theta }\right)y_{wp}(t_{\xi *},\theta_{i*}) \end{array}\right.$$

where $x_{ii_{Q_{\varepsilon }}}$ and $y_{jj_{Q_{\varepsilon }}}$ respectively represent the X and Y coordinates of the point *Q_ε_* in {W}, and Z coordinate of *Q_ε_* corresponds to the matrix element *Z*($ii_{Q_{\varepsilon }}$,$jj_{Q_{\varepsilon }}$); t_ξ_* and θ_i_* respectively represent the corresponding cutting time (s) and axial positional angle (degree) with respect to p*; as shown in Figure 8, Δ*t* and Δ*θ* are the cutting time increment (s) and the axial positional angle increment (degree) of *Q_ε_* with respect to the calculating vertex p*; and Δ*x* and Δ*y* are the coordinate increments (mm) in the X and Y directions, respectively, which can be directly calculated.

Equation (15) can be converted to

(16) $$\left\{\begin{array}{c} x_{ii_{Q_{\varepsilon }}}-x_{wp}\left(t_{\xi *},\theta_{i*}\right)=\Delta x=\left(\Delta t\frac{\partial }{\partial t}+\Delta \theta \frac{\partial }{\partial \theta }\right)x_{wp}\left(t_{\xi *},\theta_{i*}\right)\\ y_{ii_{Q_{\varepsilon }}}-y_{wp}\left(t_{\xi *},\theta_{i*}\right)=\Delta y=\left(\Delta t\frac{\partial }{\partial t}+\Delta \theta \frac{\partial }{\partial \theta }\right)y_{wp}\left(t_{\xi *},\theta_{i*}\right) \end{array}\right.$$

Δ*t* and Δ*θ* can be solved with the upper set of equations. After this, Equation (6) can be combined with (17) to calculate the height coordinates of the point Q_ε__*_ (in Figure 8, corresponding to the point *Q_ε_*) on the swept surface cut by the cutter tooth microelement in the current time interval.

(17) $$z_{Q_{\varepsilon *}}=z_{wp}\left(t_{\xi *}+\Delta t,\theta_{i*}+\Delta \theta \right)$$

Lastly, to calculate the fall-in point *Q_ε_* in the instantaneous sweeping quadrilateral of the cutter tooth microelement, from 1st to *i*th discrete point of 1st:*j*th cutter tooth in the time step 0:*t* at 1st:*q*th feed, first calculate the Z coordinate $z_{Q_{\varepsilon *}}$ of the fall-in point *Q_ε_* and compare with the stored value *Z*($ii_{Q_{\varepsilon }}$,$jj_{Q_{\varepsilon }}$). If $z_{Q_{\varepsilon *}}$ is less than *Z*($ii_{Q_{\varepsilon }}$,$jj_{Q_{\varepsilon }}$), it is indicated that the discrete cutter tooth microelement cut into the part, and *Z*($ii_{Q_{\varepsilon }}$,$jj_{Q_{\varepsilon }}$) is updated to $z_{Q_{\varepsilon }}$; otherwise, it is left unchanged. This allows for the generation of the final micro surface topography.

## 4. Experimental Validation

**Figure 9:**
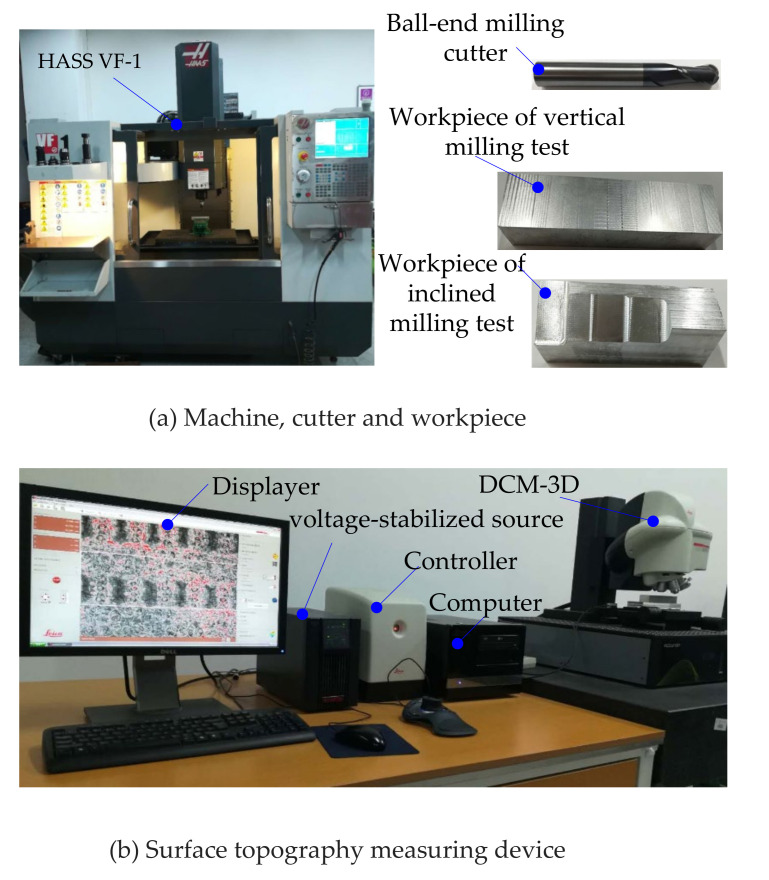
Milling and measuring device.

Trochoidal milling was performed using a high-precision CNC machine tool, Haas VF-1 vertical boring and milling high-speed machining center, and the part’s micro surface topography was measured with a Leica Laser Confocal Microscope DCM-3D at a resolution of 0.1 nm to validate the proposed model, as shown in Figure 9. The CNC system can read 20 sentences in advance, which meets the needs of trochoidal precision milling in which the trajectory is constantly changing. The generating of machining trajectory and program were performed using CAD/CAM software, CAXA Manufacturing Engineer 2018, developed in China. A new Y330 carbide ball-end-milling-cutter with diameter *ϕ*10, two cutter teeth with plane profile and elastic modulus of 210 GPa was selected as cutting tool, and its overhang length was 40 mm. The experimental part was fabricated from aerospace aluminum alloy 7050-T6 with a thickness of 35 mm; the material properties are shown in Table 1, where: *E* is elastic modulus at room temperature, *ρ* is material density, *c* is specific heat, *λ* is thermal conductivity, *δ_l_* is linear expansion coefficient, and *μ* is Poisson’s ratio. The cutter wear was ignored as the workpiece material was easy to cut, the tool material was wear-resistant, and the experiment time was very short. Cutting parameters of the experiment are shown in Table 2.

In order to determine the consistencies between the simulated and experimental micro surface topographies, both of their three-dimensional micro surface topographies were compared, and the differences in two-dimensional sectional profiles of micro surface topographies both in the leading feed direction and the interval feed direction were tested using two-sided hypothesis testing method of the paired data (*t*-test method). If the data of both the simulated and experimental sectional profiles satisfied Equation (18), the simulated data could be used for comprehensive evaluation of actual processed surfaces. In addition, both arithmetic mean deviations *R*_a_ of the sectional profiles were calculated, and the relative error between both was calculated in order to directly illustrate the simulation accuracy.

(18) $$\left|t|=\left|\frac{\bar{d}}{S_{D}/\sqrt{n_{s}}}\right|<t_{\gamma /2}(n_{s}-1\right)$$

where $\bar{d}$ and *S_D_* are a sample mean and sample standard deviation of the difference between simulation and experiment, respectively; and *n_s_* and *γ* are sample size and test level, respectively. In the experiment, *n_s_* and *γ* were set to 71 and 0.1, respectively, so $t_{\gamma /2}(n_{s}-1)$= *t*_0.05_(70) = 1.667. If |*t*| < 1.667, it indicates no significant differences.

**Figure 10:**
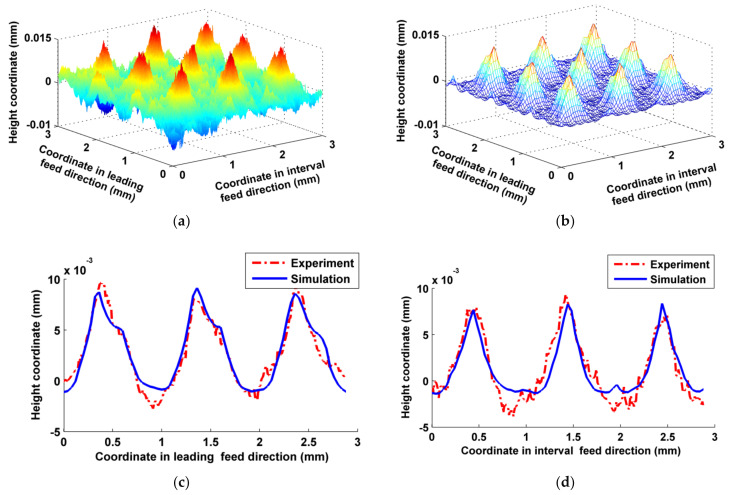
Comparisons between the simulated and experimental micro surface topography in inclining milling. (**a**) Actual milled surface topography; (**b**) Simulated surface topography; (**c**) Comparison of the sectional profiles in leading feed direction; (**d**) Comparison of the sectional profiles in interval feed direction.

In the inclining milling test, the three-axis interlocking interpolation method was used for trochoidal milling on an inclined surface. As shown in Figure 10, three-dimensional micro surface topographies of the simulated and the actual milled were close overall. The simulated sectional profiles in both directions were also basically consistent with the corresponding actual milled ones apart from certain differences in the valley. The main reason for these differences was that the metal in the valley of the milled surface was subjected to more extrusions, and therefore was prone to plastic deformation and plastic flow. As the trochoidal milling trajectories overlapped with each other, and the entry and exit directions of the cutter teeth were always changing, the velocity fields of the metal deformation and flow in this area were relatively disordered. Trochoidal milling is a hybrid milling method that involves down milling, up milling, push milling and pull milling [19], and these different milling modes cause various transformations during processing, which can easily cause cut chips to stick on the part’s surface. According to the principle of cutting, an area of a part in the X_w_O_w_Y_w_ plane may be acted upon by multiple effects, such as different cutting trajectories, different cutter teeth and different parts of same cutter tooth, etc., which result in the occurrence of chip-less or little-chipped back-cutting. If the back-cut thickness does not reach the critical thickness of the smallest cutting layer of the metal to be cut, the cutter teeth only exert scratching and squeezing forces rather than cutting on the cut surface, and this causes random wrinkles on the surface. From comparing Figure 10c,d, there is a greater difference at the bottom of profile in the valley in the interval feed direction. The concave valley next to the convex peak on the surface was mainly machined by the current feed trajectory of cutter teeth in the leading feed direction, whereas in the interval feed direction, in addition to the effect of the current feed trajectory, there were many effects of adjacent feed trajectories, which meant that there was a higher frequency of back-cutting on the same part of the surface, resulting in more wrinkles. The calculated values of |*t*| in the sectional profiles shown in Figure 10c,d corresponding to leading feed direction and interval feed direction were 1.0776 and 0.2288, respectively, which both satisfied Equation (18). According to Table 3, the differences in *R_a_* between the simulated and experimental profile in both directions were small.

**Figure 11:**
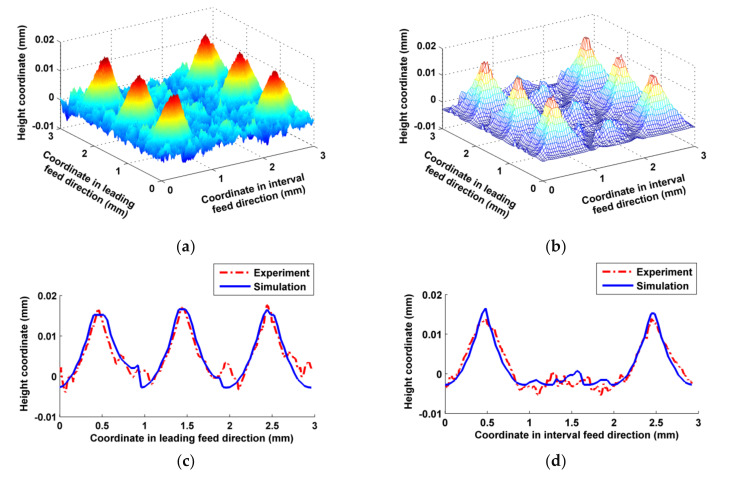
Comparisons between the simulated and experimental micro surface topography in vertical milling. (**a**) Actual milled surface topography; (**b**) Simulated surface topography; (**c**) Comparison of the sectional profiles in leading feed direction; (**d**) Comparison of the sectional profiles in interval feed direction.

In the vertical milling test, the peak shapes of both surface topographies were very similar, as shown in Figure 11; the differences in the valley were mainly attributable to the characteristics of ball-end vertical milling: the velocity of the cutter’s bottom is low, and the leading effects of squeezing and ploughing cause the part’s material to undergo plastic flow and deformation. Cutter wear, especially in the ball head, may also have a certain effect on the formation of micro surface topography [44]. There were some differences near the bottom valleys of the sectional profiles shown in Figure 11c, because the larger slippage and plastic flow of the metal cutting layer mainly appeared in the leading feed direction. However, the differences between the sectional profiles of the actual milled and simulated topographies were smaller, as shown in Figure 11d, as the cutting effect of the cutter on the metal cutting layer was improved, while the extrusion effect was weakened in the interval feed direction. |*t*| of the sectional profiles in Figure 11c,d were 1.4446 and 0.3736, respectively, and both satisfied Equation (18). *R_a_* of the actual milled and simulated surface at the corresponding direction were close overall, as shown in Table 4. However, there was a certain gap in accuracy relative to the simulation results of the inclined milling test, due to the reasons mentioned above.

Therefore, ball-end trochoidal milling micro surface topography simulated by this method was largely consistent with the actual milled results, and so can be used for the prediction of micro surface topography.

## 5. Simulation Analysis

To illustrate the influence of trochoidal milling parameters on the micro surface topography, surface characterization comparisons were made in two aspects: amplitude and function (including some specific functional properties such as bearing, sealing, lubricant retention capabilities, etc.). Parameters such as arithmetic mean deviation *S_a_*, maximum height *S_z_*, skewness *S_sk_* and kurtosis *S_ku_* were selected to characterize the amplitude of the surface, and parameters such as center oil storage coefficient *S_ci_*, bottom oil storage coefficient *S_vi_*, peak load bearing coefficient *S_bi_*, and center bearing coefficient *S_bc_* were used to characterize the function of the surface; *S_ci_* and *S_vi_* are calculated according to the formula given in [45].

### 5.1. Influence of the Amplitude of the Trochoid on the Micro Surface Topography

**Figure 12:**
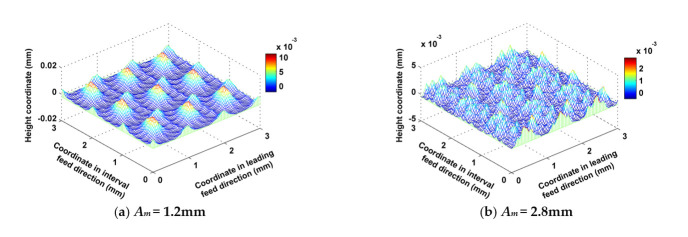
Ball-end milling micro surface topography comparison between different amplitudes of the trochoid.

**Figure 13:**
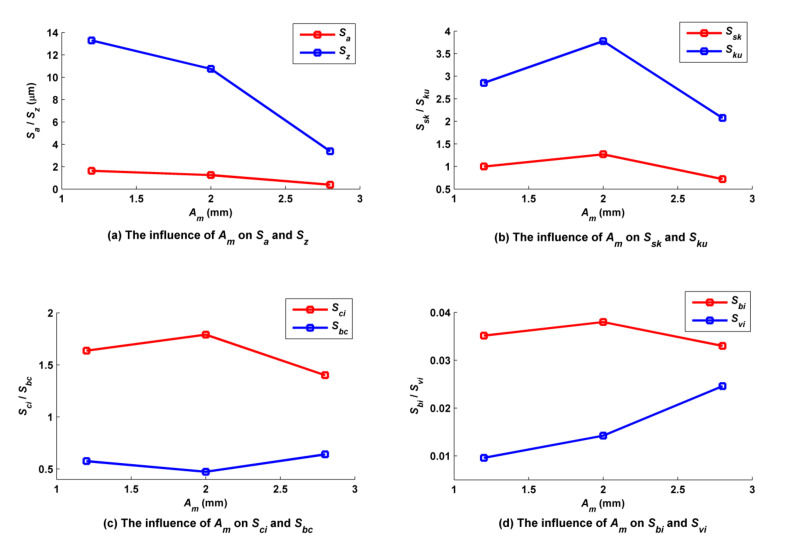
Influence of amplitude on ball-end trochoidal milling surface characteristics.

The amplitude of the trochoid *A_m_* was set to 1.2, 2.0 and 2.8 mm, while other cutting parameters such as *f_f_*, *f_p_*, *a_p_*, *N*, *α*, *β* and *τ* were kept at 0.2 mm/z, 1 mm, 0.5 mm, 6000 rpm, 30°, 33.7° and 1 mm respectively. As shown in Figure 12, when *A_m_* was small (1.2 mm), there were big main peaks, and a large quantity of annular grooves formed by the trochoidal trajectory around these peaks. The regularity of the distribution of convex peaks and grooves is obvious. However, when *A_m_* was great (2.8 mm), there were many small peaks which were formed by back cutting, and they were randomly distributed. As Figure 13a shows, both *S_z_* and *S_a_* decreased as *A_m_* increased, because the cutter’s engagement with the part grew wider as *A_m_* increased, and the overlapping areas of any two adjacent trajectory rows became wide accordingly, so that residual volumes were back cut or separated by the adjacent trajectory rows. The rules by which *A_m_* influences *S_sk_* and *S_ku_* are shown in Figure 13b. *S_sk_* was greater than 0, which indicated that the obtained surfaces under these three cutting conditions all belonged to the category of surfaces with many peaks and few valleys, but the number of convex peaks decreased as *A_m_* increased when *A_m_* was greater than 2.0 mm, and the number of surface scallops increased accordingly. Three values of *S_ku_* were close to three when *A_m_* was less than 2.0 mm, and *S_ku_* reached a maximum when *A_m_ was* equal to 2 mm, which indicated that the convex peaks and scallops of these three surfaces were all steep, the steepness peaking when *A_m_* was equal to 2 mm and then subsequently decreasing. The rules by which *A_m_* influences *S_ci_* and *S_bc_* are shown in Figure 13c. *S_ci_* of all these three surfaces was great and close to the typical value (1.56) of a surface following a Gaussian distribution, which indicated that the oil storage rates of the three surfaces in their center regions were better. *S_ci_* reached a maximum when *A_m_* was equal to 2 mm, i.e., the best capacity of oil storage was reached at this condition. However, surface center bearing capacity displayed the opposite trend, as shown in *S_bc_*. The rules by which *A_m_* influences *S_bi_* and *S_vi_* are shown in Figure 13d. The values of *S_bi_* and *S_vi_* were both small when *A_m_* was set at different values; the bearing stiffness of the surface peaks increased as *A_m_* increased, but the oil storage performance of the surface bottom valley deterioriated when *A_m_* was greater than 2 mm.

### 5.2. Influence of the Pitch of the Trochoid on the Micro Surface Topography

**Figure 14:**
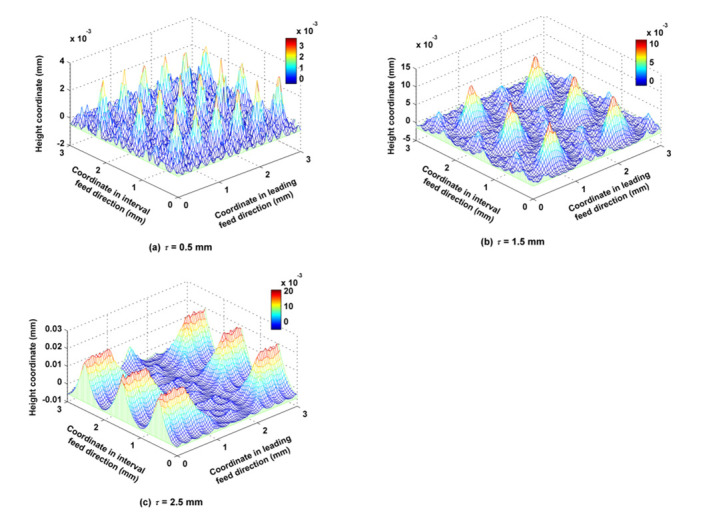
Ball-end milling micro surface topography comparisons among different pitches of the trochoid.

**Figure 15:**
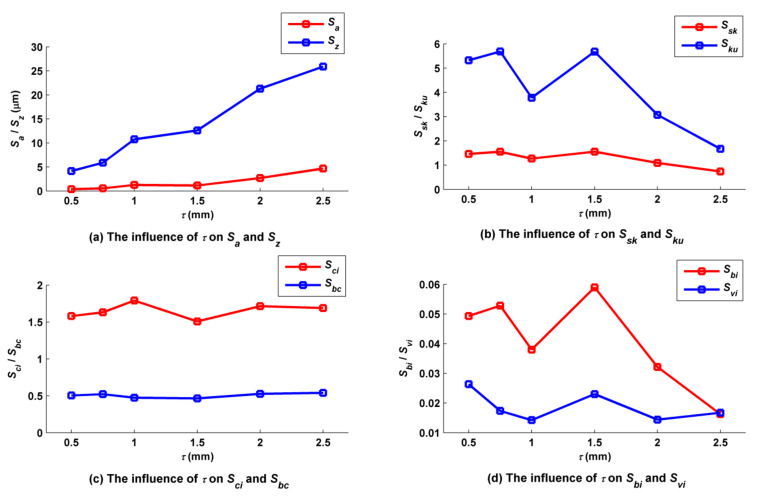
Influence of pitch on ball-end trochoidal milling surface characteristics.

The pitch of the trochoid *τ* was set to 0.5, 0.75, 1.0, 1.5 and 2.0 mm, while the other cutting parameters such as *f_f_*, *f_p_*, *a_p_*, *N*, *α*, *β* and *A_m_* were kept at 0.2 mm/z, 1 mm, 0.5 mm, 6000 rpm, 30°, 33.7° and 2 mm, accordingly. According to Figure 14, the main peaks of the micro surface topographies were similar to’stalactite’ shapes, and the number of these shapes in a unit area decreased with increases in *τ*. For example, when *τ* was equal to 0.5 mm, there were 18 ’stalactites’ within the range of 3 mm × 3 mm, whereas there were only six when *τ* was equal to 1.5 mm. However, the main peaks of the micro surface topography were in the shape of a ’short ridge’ when *τ* increased to 2.5 mm. In fact, both ’stalactite’ and ’short ridge’ shapes are triangular pyramids; when *τ* was small, the length of the triangular pyramid in the leading feed direction was small. As shown in Figure 15a, because the length of residual convex body of surface in the leading feed direction was increased, its height increased with increases in *τ*, and both *S_z_* and *S_a_* increased with increases in *τ*. The relationships of *S_sk_* and *S_ku_* to *τ* are shown in Figure 15b. The machined surface had many peaks and few valleys; these peaks and valleys were steep when *τ* was less than 2 mm, and therefore *S_sk_* and *S_ku_* were greater than 0 and 3, respectively. However, the number and steepness of peaks both decreased when *τ* exceeded 1.5 mm. The relationships of *S_ci_* and *S_bc_* to *τ* are shown in Figure 15. The oil storage capacity of the surfaces in their center regions fluctuated with *τ*; however, when *τ* was equal to 1.5 mm, this capacity decreased to the minimum because of the increased convex peaks and their relatively steep characteristics. Surface center region bearing capacity remained basically unchanged with changes in *τ*. The relationships of *S_bi_* and *S_vi_* to *τ* are shown in Figure 15d. The overall bearing capacity of the upper surface summit decreased with increases in *τ*; however, when *τ* was equal to 1.5 mm, abnormal mutation occurred as the bearing area of the surface summit increased due to increased convex peaks and relatively steep slopes. The oil storage capacity of the surface valley bottom fluctuated with increases in *τ* and then decreased.

### 5.3. Influence of Feed per Tooth on the Micro Surface Topography

**Figure 16:**
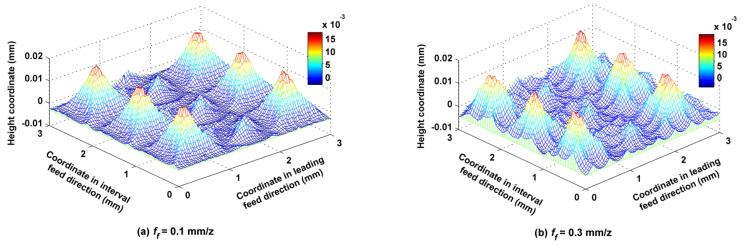
Ball-end trochoidal milling micro surface topography comparisons between different feeds per tooth.

**Figure 17:**
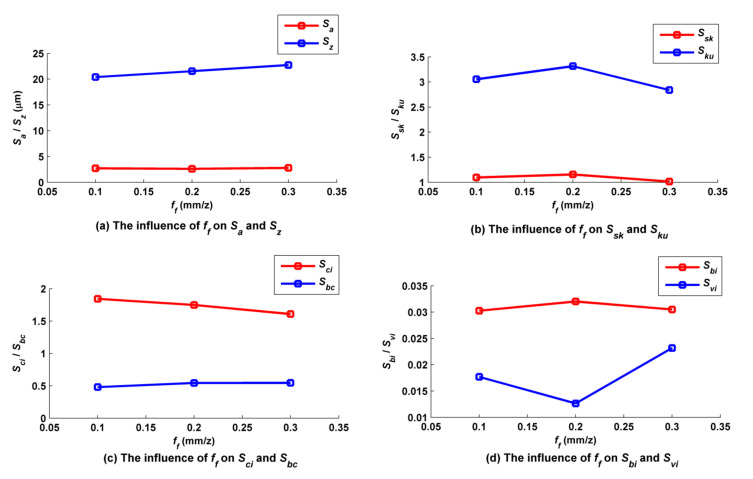
Influence of feed per tooth on ball-end trochoidal milling surface characteristics.

Feed per tooth *f_f_* was set to 0.1, 0.2, and 0.3 mm/z, while the other cutting parameters such as *f_p_*, *a_p_*, *N*, *α*, *β*, *A_m_*, and *τ* were kept at 1.0 mm, 0.5 mm, 6000 rpm, 30°, 33.7°, 2 mm and 2 mm, accordingly. According to Figure 16, *f_f_* had little effect on the main peaks of the surface, but had a great effect on the secondary peaks; for example, the secondary peaks of the surface when *f_f_* was equal to 0.3 mm/z were steeper than those when *f_f_* was equal to 0.1 mm/z. As shown in Figure 17a, *S_a_* basically did not change with changes in *f_f_*, but *S_z_* slightly increased with increases in *f_f_*, because the varying of *f_f_* has much less influence on the residual height of the surface than the trochoidal trajectory does. The relationships of *S_sk_* and *S_ku_* to *f_f_* are shown in Figure 17b. There were always many more peaks and fewer valleys on the surface under the three different values of *f_f_*, but the number of convex peaks decreased as *f_f_* increased, and the steepness of the surface peaks first increased and then decreased as *f_f_* increased. The relationships of *S_ci_* and *S_bc_* to *f_f_* are shown in Figure 17c. The oil storage capacity of the surfaces in their center regions overall decreased with increases in *f_f_*, but bearing capacity was basically independent of *f_f_*. The relationships of *S_bi_* and *S_vi_* to *f_f_* are shown in Figure 17d. The overall bearing capacity of the upper surface summit slightly fluctuated with increases in *f_f_*, and the corresponding surface abrasion capacity remained basically unchanged. *S_vi_* decreased with increases in *f_f_* at first, and then increased faster, indicating that an appropriate increase in *f_f_* is conducive to improving oil storage performance at the bottom of the surface valley.

### 5.4. Influence of Stepover on the Micro Surface Topography

**Figure 18:**
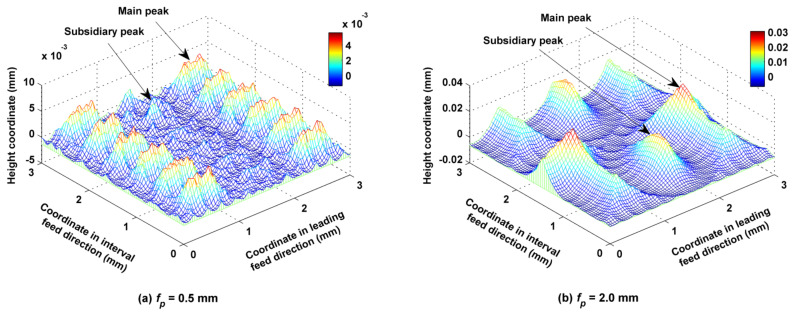
Ball-end trochoidal milling micro surface topography comparisons between different leading stepovers.

**Figure 19:**
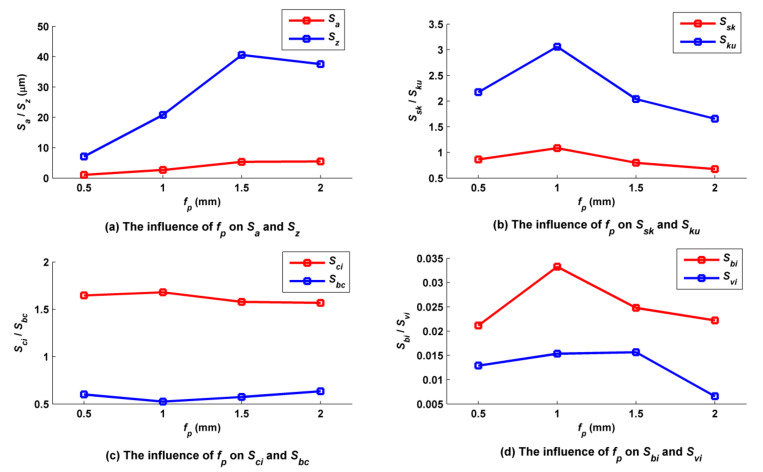
Influence of the leading stepover on ball-end trochoidal milling surface characteristics.

Stepover *f_p_* was set to 0.5, 1.0, 1.5 and 2.0 mm, while the other cutting parameters such as *f_f_*, *a_p_*, *N*, *α*, *β*, *A_m_* and *τ* were kept at 0.2 mm/z, 0.5 mm, 6000 rpm, 30°, 33.7°, 2 mm and 2 mm, accordingly. As shown in Figure 18, the volumes and heights of both the main and secondary peaks of the surface increased as *f_p_* increased from 0.5 mm to 2 mm. According to Figure 19a, with the increase in *f_p_*, *S_z_* rose with it in the first phase, but there was a twist at *f_p_* = 1.5 mm as interaction capacity between two adjacent trajectory rows was strengthened. *S_a_* increased with increases in *f_p_* because the residual height became larger. According to Figure 19b, the characteristic of many peaks and few valleys on the surface did not change overall when *f_p_* was adjusted, so that all *S_sk_* values were positive; however, the number and steepness of convex peaks reached a maximum at *f_p_* = 1.0 mm, and subsequently decreased. According to Figure 19c, the oil storage capacity of the surfaces in their center regions was overall the best when *f_p_* was equal to 0.5 mm and 1 mm, because a small *f_p_* could keep the main concave valleys on the surface from being connected, therefore ensuring good oil storage capacity. The bearing capacity of the surfaces in their center regions improved overall with increases in *f_p_* because the bearing area of these regions increased. According to Figure 19d, when *f_p_* was lower than or equal to 1.0 mm, increases in *f_p_* caused the numbers of surface peaks to increase, so that the bearing area of surface summit increased and *S_bi_* increased, and the run-in capacity of surface was improved. However, when *f_p_* was greater than 1.0 mm, the volume of a single surface convex body decreased with any increase in *f_p_*, and *S_bi_* decreased. At first, surface bottom valley volume decreased with increases in *f_p_*, but after a certain point, the influence of *f_p_* on the volume became smaller; *S_vi_* decreased with increases in *f_p_* to begin with and then remained at a low level, which means that decreases in *f_p_* were conducive to improving the oil storage performance at the bottom of the surface.

### 5.5. Influence of Lead Angle on the Micro Surface Topography

**Figure 20:**
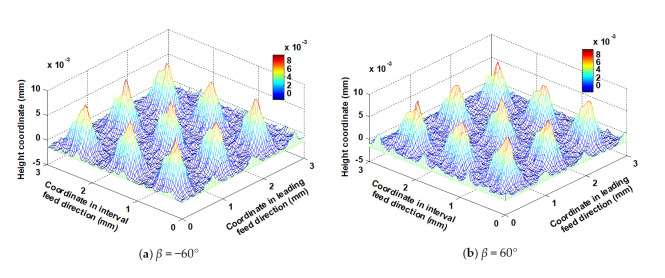
Ball-end trochoidal milling micro surface topography comparisons between different lead angles.

**Figure 21:**
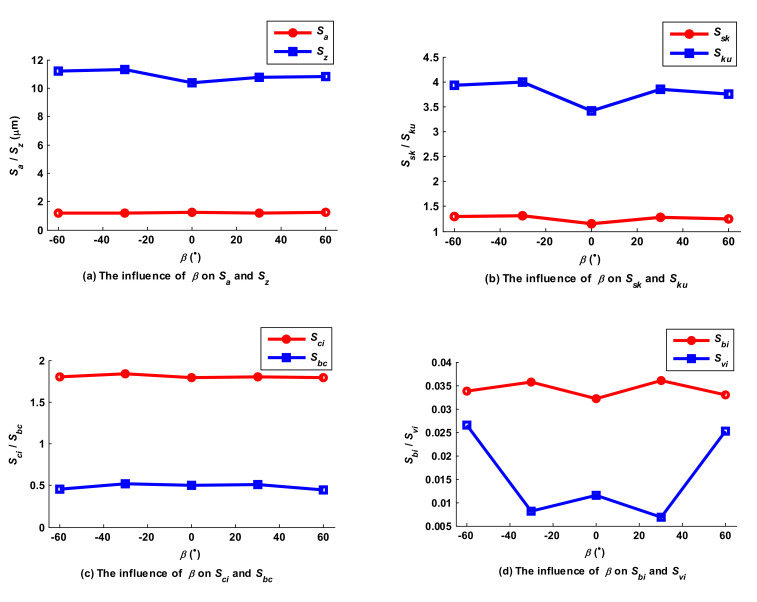
Influence of the lead angle on ball-end trochoidal milling surface characteristics.

Lead angle *β* was respectively set to −60°, −30°, 0°, 30° and 60°, while other parameters such as *f_p_*, *f_f_*, *a_p_*, *N*, *α*, *A_m_* and *τ* were kept at 1 mm, 0.2 mm/z, 0.5 mm, 6000 rpm, 0°, 2 mm and 2 mm, respectively. Figure 20 shows the shapes of the micro surface topographies corresponding to *β* equal to −60° and 60°, respectively. Both were similar because the lead angle has less effect on the shape of micro surface topography than the trochoidal trajectory, which accounts for the relationship between *S_a_* and *β* shown in Figure 21a: *S_a_* basically did not change with the change in *β*. There were certain effects of *β* on *S_z_*; the reasons are as follows: regardless of cutter inclination to any particular extent, the depth coordinates of the bottom of the micro surface topography are constant, but the surface main peak height is not constant, which has a direct effect on *S_z_*. When the current lead angle is positive, the area around the main peak tends to be subject to down milling by the cutter; by contrast, when the current lead angle is negative, the same area tends to be cut by up milling. From the perspective of the geometric relationship, the residual heights are higher, and the radius of the cutter’s action upon the ring of the surface main peak is smaller when the cutter is inclined at a lead angle compared with inclination at 0° (vertical milling), and therefore more likely to leave a large residual height. As shown in Figure 21b, the surface was characterized by many peaks and fewer valleys, and the number of convex peaks corresponding to the surface was less than in other cases, because the residual convex area left by one cutter tooth was cut off by the back of the next cutter tooth. The surface convex peaks were steep, and therefore *S_ku_* was greater than 3. When *β* was 0°, the steepness of the convex peak was the smallest. When *β* was set to −30°, the cutter radius acting on the surface of the main peak was smaller than when *β* was set to −60°; therefore the residual height was higher, and the steepness of the convex peak was the largest. This feature also affected the oil storage capacity and bearing capacity of the central region of the surface, as shown in Figure 21c; *S_ci_* and *S_bc_* were the largest when *β* = −30°. In Figure 21d, the bearing area of the surface peak was the largest and hence *S_bi_* was the greatest when *β* was equal to −30°; the connectivity of the bottom of the surface valley was relatively good at this value, so that the oil storage capacity was not good and the *S_vi_* was the smallest.

### 5.6. Influence of Tilt Angle on the Micro Surface Topography

**Figure 22:**
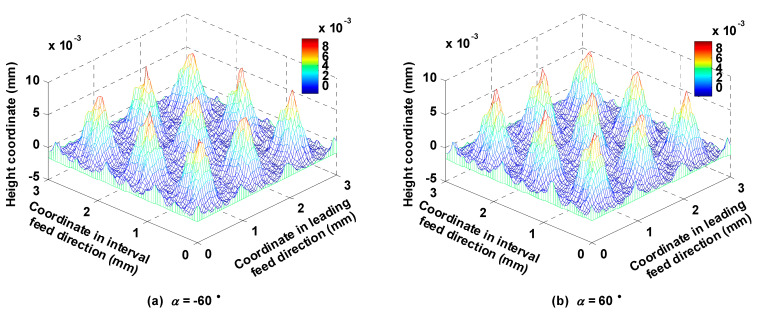
Ball-end trochoidal milled micro surface topography comparison between different tilt angles.

**Figure 23:**
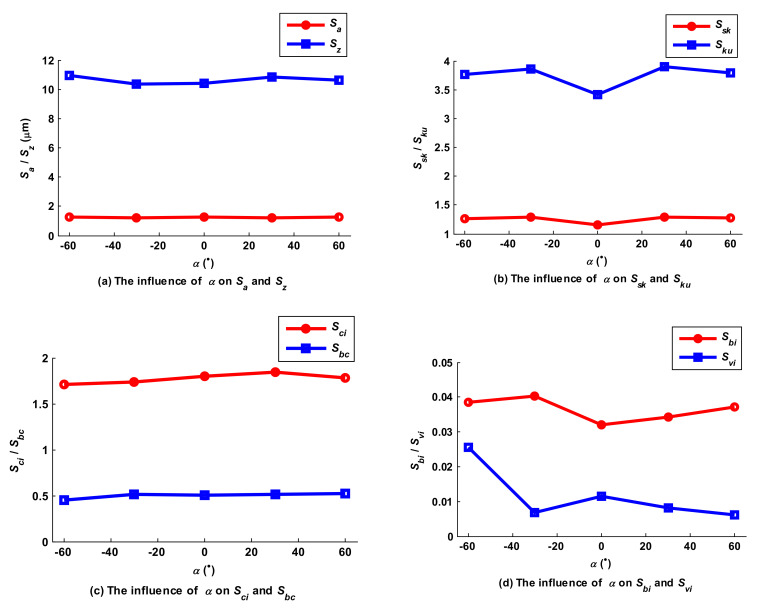
Influence of the tilt angle on Ball-end trochoidal milled surface characteristics.

Tilt angle *α* was set to −60°, −30°, 0°, 30° and 60°, while the other parameters such as *f_p_*, *f_f_*, *a_p_*, *N*, *β, A_m_* and *τ* were set to 1mm, 0.2 mm/z, 0.5 mm, 6000 rpm, 0°, 2 mm and 2 mm, respectively. In Figure 22, the shape of the micro surface topography was similar when *α* was equal to −60° and 60°, because, as with the lead angle, tilt angle has less effect on the shape of micro surface topography relative to trochoidal trajectory, which accounts for the relationship between *S_a_* and *α*, shown in Figure 23a: *S_a_* basically did not change with the change in *α*. However, the influence curve of *α* on *S_z_* was wavy. According to the projection view, the cutting state of the lower side of the surface main peak was different when the cutter’s tilting posture was different; for example, down milling occurred and *S_z_* reached a maximum when *α* was equal to 30°, but up milling occurred and *S_z_* reached a minimum when *α* was equal to −30°. According to Figure 23b, adjusting tilt angle could not completely change the overall features of the milling surface, which was characterized by many steep peaks and few valleys, so that *S_sk_* and *S_ku_* were greater than 0 and 3, respectively. When *α* increased from −60° to −30°, the number of surface peaks basically remained unchanged, and *S_sk_* was therefore basically constant. However, the steepness of the convex peaks of the surface was increased, leading *S_ku_* to increase slightly because of cutter acting radius reduction and down milling. When *α* increased from −30° to 0°, the number of valleys and peaks slightly increased and then decreased, combining with the acting radius of the cutter on the side of surface convex peaks increasing as a whole, so that the degree of surface peak steepness decreased and *S_ku_* decreased rapidly. According to Figure 23c, when *α* increased from −60° to 30°, cutter acting radius constantly increased overall and led to the cubage of the surface valleys increasing; *S_ci_* increased gradually, but remained almost unchanged when *α* increased from 30° to 60° because the cutter acting radius was almost constant. However, when *α* increased from −60° to −30°, the number of micro convex bodies in the center region of the surface increased, and *S_bc_* increased accordingly; subsequently, it basically remained constant when *α* increased from −30° to 60°. As shown in Figure 23d, when *α* increased from −60° to −30°, the bearing area of the surface peaks increased slightly and surface valleys became relatively smooth, so that *S_bi_* increased slightly and *S_vi_* accordingly decreased dramatically. However, the bearing area of the surface peaks decreased and the scalloped area in the surface valley increased when *α* increased from −30° to 0°, so that *S_bi_* and *S_vi_* decreased and increased, accordingly. This tendency became reversed when *α* increased from 0° to 60°; the bearing area of the surface peaks increased gradually and scalloped area in the surface valley decreased gradually, and therefore *S_bi_* increased and *S_vi_* decreased accordingly.

## 6. Conclusions

This paper proposes a Z-MAP method for the modeling of ball-end trochoidal milling micro surface topography, based on which a comparison of surface characteristics between the results of ball-end trochoidal milling and linear milling was conducted, and the rules by which cutting parameters of the trochoidal milling influence the surface characteristics were revealed. The following conclusions were reached:

1. The modeling of ball-end trochoidal milling micro surface topography is based on the following assumptions: the trajectory of the cutter tooth can be established by homogeneous coordinate matrix transformation; the part, cutter and milling time are reasonably discrete; the fall-in points of the part are detected through follow-up matrix encirclement and the angle accumulation method; and the height coordinates of the fall-in points are calculated by a Taylor formula-based interpolation method. The simulation and experiment results had good agreement on three-dimensional micro surface topographies, and the differences in *R_a_* in the sectional profiles were less than 10% in both vertical milling and inclined milling. The *t*-test values of these sectional profiles all satisfied |*t*| < 1.667, which means there were no significant differences between the simulation and experiment. Hence, this model can be used to substitute for experiments to research ball-end trochoidal milling micro surface topography.
2. The rules governing the influence of cutting parameters, such as amplitude and pitch of the trochoid, feed per tooth, stepover, cutter lead angle and tilt angle, on the amplitude and functional parameters of micro surface topography were derived from the simulated data. Unlike in common milling modes, amplitude and pitch of the trochoid and stepover were the main factors which influenced the resulting surface characteristics.

## Figures and Tables

**Table 1:** Characteristics of the 7050-T6.

Material	*E* (GP_a_)	*μ*	*ρ* (kg/m^3^)	*c* (J/(kg∙m))	*λ* (W/(m∙K))	*δ_l_* (10^−6^/K)
7050-T6	69	0.30	2850	526	114	20.8

**Table 2:** Milling parameters for validation.

Milling Parameters	*N* (rpm)	*f_f_* (mm/z)	*f_p_* (mm)	*a_p_* (mm)	*A_m_* (mm)	*τ* (mm)	*α* (º)	*β* (º)
Setting in inclining milling	6000	0.2	1	0.5	2	1	0	30
Setting in vertical milling	6000	0.2	1	0.5	2	2	0	0

**Table 3:** *R_a_* of the experimental and simulated sectional profiles of the micro surface topography.

Sectional Profile	*R_a_* of the Experiment (µm)	*R_a_* of the Simulation (µm)	Relative Error (%)
In leading feed direction	2.78	2.94	5.75
In interval feed direction	2.52	2.41	4.37

**Table 4:** *R_a_* of the experimental and simulated sectional profiles obtained by vertical milling.

Sectional Profile	*R_a_* of the Experiment (µm)	*R_a_* of the Simulation (µm)	Relative Error (%)
In leading feed direction	4.69	5.11	8.96
In interval feed direction	4.76	4.43	6.93

## Data Availability

Not applicable.
